# Directed Differentiation of Human Induced Pluripotent Stem Cells into Fallopian Tube Epithelium

**DOI:** 10.1038/s41598-017-05519-2

**Published:** 2017-09-06

**Authors:** Nur Yucer, Marie Holzapfel, Tilley Jenkins Vogel, Lindsay Lenaeus, Loren Ornelas, Anna Laury, Dhruv Sareen, Robert Barrett, Beth Y. Karlan, Clive N. Svendsen

**Affiliations:** 1 Board of Governors Regenerative Medicine Institute, Cedars-Sinai Medical Center, Los Angeles, CA 90048 USA; 2 Women’s Cancer Program, Samuel Oschin Comprehensive Cancer Institute, Cedars-Sinai Medical Center, Los Angeles, CA 90048 USA; 3 Department of Pathology and Laboratory Medicine, Cedars-Sinai Medical Center, Los Angeles, CA 90048 USA; 4 Department of Biomedical Sciences, Cedars-Sinai Medical Center, Los Angeles, CA 90048 USA

## Abstract

The fallopian tube epithelium (FTE) has been recognized as a site of origin of high-grade serous ovarian cancer (HGSC). However, the absence of relevant *in vitro* human models that can recapitulate tissue-specific architecture has hindered our understanding of FTE transformation and initiation of HGSC. Here, induced pluripotent stem cells (iPSCs) were used to establish a novel 3-dimensional (3D) human FTE organoid *in vitro* model containing the relevant cell types of the human fallopian tube as well as a luminal architecture that closely reflects the organization of fallopian tissues *in vivo*. Modulation of Wnt and BMP signaling directed iPSC differentiation into Müllerian cells and subsequent use of pro-Müllerian growth factors promoted FTE precursors. The expression and localization of Müllerian markers verified correct cellular differentiation. An innovative 3D growth platform, which enabled the FTE organoid to self-organize into a convoluted luminal structure, permitted matured differentiation to a FTE lineage. This powerful human-derived FTE organoid model can be used to study the earliest stages of HGSC development and to identify novel and specific biomarkers of early fallopian tube epithelial cell transformation.

## Introduction

High-grade serous carcinoma (HGSC), the most common subtype of epithelial ovarian cancer (~70%), has the highest mortality rate among all gynecological cancers^1^. HSGC patients have poor prognoses due to a combination of factors including late stage at diagnosis and a high predilection for developing drug resistance. It is now accepted that the majority of HGSCs arise from the secretory cells of the fallopian tube epithelium (FTE). The discovery of *in situ* lesions in the fallopian tube fimbriae, namely serous tubal intraepithelial carcinoma (STIC), supports the concept of the FTE origin of serous “ovarian” carcinoma^2–6^. The discovery of an extra-ovarian origin of ovarian cancer is a fundamental advance toward improving early detection, prevention and treatment of this lethal disease. However, the lack of relevant *in vitro* human models that can recapitulate tissue-specific architecture and study early alterations has hindered the further understanding of FTE transformation as well as the initiation and progression of HGSC.

Fallopian tube epithelium is composed of a polarized columnar epithelium including ciliated and secretory cells. Current fallopian tube models, including *ex vivo* and three-dimensional (3D) spheroid models, demonstrate the importance of cell polarity in recreating secretory and ciliated cells^7–9^. However, FTE cells in these models have a reduced proliferation rate and induced senescence because of the lack of convoluted luminal architecture and the ectopic microenvironment^7–9^. Mouse models, including patient-derived xenografts and genetically engineered mice, have overcome some of these limitations and yielded significant insights into the basis of cancer development^10–14^. However, the complexity of a human tumor is not reliably represented in mouse models. Furthermore, it has been challenging to engineer silent and expressed mutations with the correct expression timeframe as well as accurate targeting to specific tissue and cell types, such as the secretory cell of the fallopian tube.

Alternatively, induced pluripotent stem cell (iPSC) technology and 3D-tissue engineering provide powerful tools to recapitulate physiologically relevant aspects of disease progression *in vitro*. iPSCs are generated by reprogramming somatic cells and can be subsequently differentiated into various cell types^15, 16^. Recently, patient-derived iPSCs have been used to model several inherited human diseases and to successfully generate relevant cell types that display disease pathogenesis^17–20^. However, remaining challenges with iPSC-based modeling include establishing direct differentiation protocols for desired cell types and integrating the differentiated cells into functional tissue structures.

Here, we describe a rapid and efficient method to create an iPSC-derived 3D model of human FTE with the desired cell types and luminal architecture. The female reproductive tract, including FTE, arises from the Müllerian duct in parallel to the urinary system from intermediate mesoderm (IM) of the urogenital ridge in the posterior primitive streak. We have recapitulated Müllerian development *in vitro* by inducing IM differentiation and further developed FTE precursors by adding pro-Müllerian growth factors. Correct differentiation was monitored through the expression of cell-related markers such as *PAX2, GATA3, OSR1, WT1*, and *OVGP1* at each step. Further FTE lineage differentiation was obtained on a 3D growth platform, which enabled the FTE organoid to self-organize into a convoluted luminal structure. Importantly, staining of secretory and ciliated cellular components demonstrated that these structures accurately model fallopian tube architecture.

## Results

### Robust Differentiation of Human iPSCs Into Intermediate Mesoderm-Like Cells

**Figure 1:**
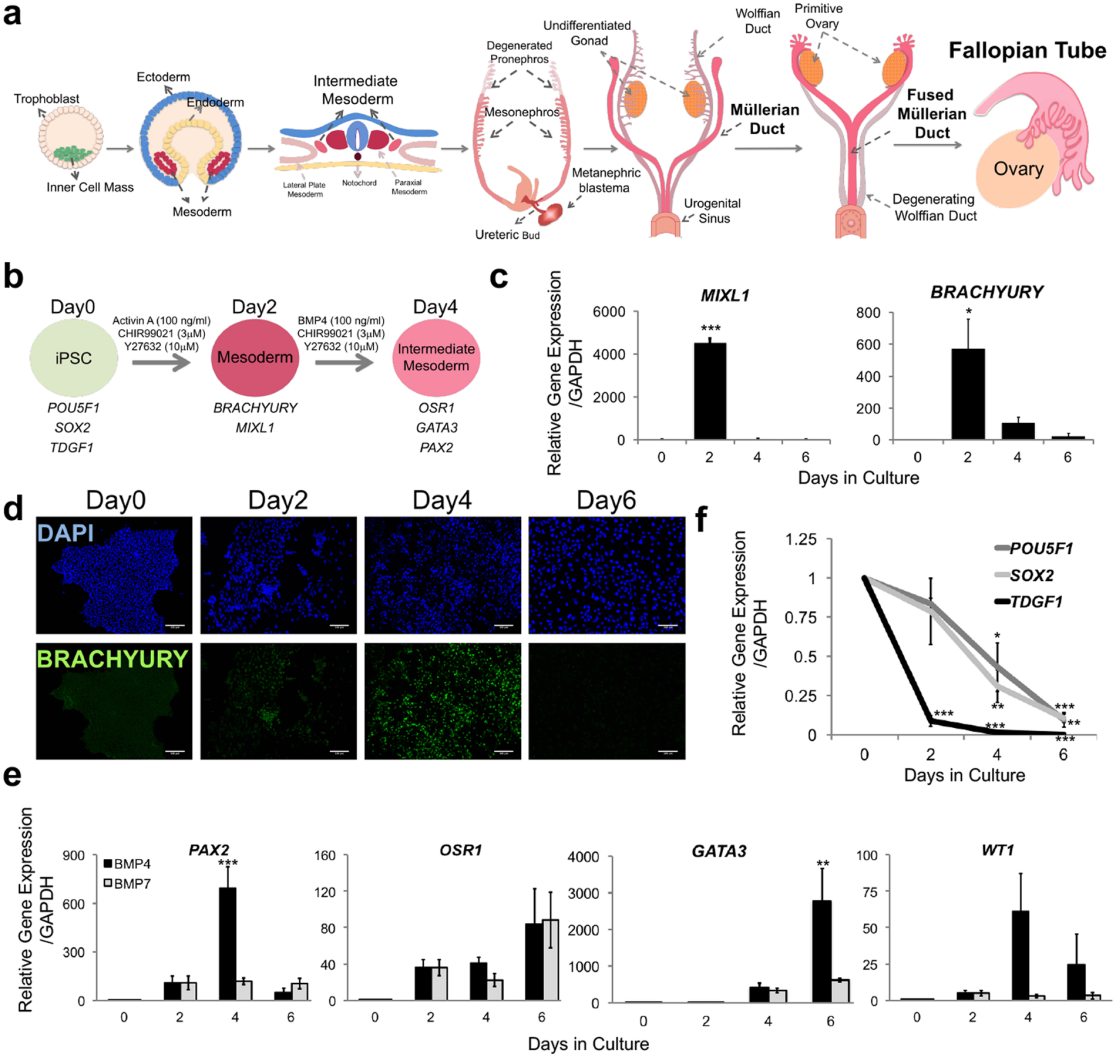
Differentiation of Human iPSC into Intermediate Mesoderm-Like Cells. (**a**) Schematic of developmental stages from the inner cell mass to female reproductive tract and fallopian tube. (**b**) Timeline and factors involved in the differentiation of iPSCs into intermediate mesoderm. (**c**) Expression kinetics of mesoderm markers *MIXL1*- and *BRACHYURY* during the 6-day differentiation course using Mae *et al*., protocol^21^. (**d**) Immunocytochemistry demonstrating expression and localization of mesoderm marker BRACHYURY during differentiation. (**e**) Expression of intermediate mesoderm markers, *PAX2, OSR1, GATA3* and *WT1* during the 6-day differentiation course. (**f**) Pluripotency markers *POU5F1, SOX2* and *TDGF1* expression during the 6-day differentiation course. Relative gene expression to iPSC stage (day 0) was calculated using ΔΔCt method and normalized to endogenous *GAPDH* level for 87iCTR-n3 iPSC line. Error bars are Standard Error of the Mean (SEM) (n = 3 independent biological experiments). One way ANOVA with Holm-Sidak post-hoc test was used for this analysis, with significance at *p ≤ 0.05, **p ≤ 0.01, ***p ≤ 0.001.

The mesoderm differentiates into the IM in a sequential manner determined by the differential expression of specific transcription factors in response to signaling pathways, including WNT, Nodal/Activin and Bone Morphogenetic Proteins (BMPs). The IM subsequently develops in parallel into both the fallopian tube and kidney in humans (Fig. 1a,b). At 6 weeks post-fertilization, mesonephric ducts are formed and develop separately into the Müllerian ducts. There are several protocols for kidney development, which primarily utilize signaling molecules in various temporal combinations^21–23^. However, no successful protocol exists, to date, for iPSC development into the fallopian tube epithelial cells.

In order to create a specific 3D iPSC-derived model of FTE and study the cell of origin effect on FTE differentiation, we used three different female human iPSC lines (87iCTR-n3, 01iMEC-n4, 14iCTR-n6) that were reprogrammed initially from B-cells (from blood), mammary epithelial cells (from mammary epithelium biopsy), and fibroblasts (from skin biopsy), respectively (S1 Table). Each line demonstrated the molecular hallmarks of pluripotent cells, including morphology, normal karyotype, expression of alkaline phosphatase and pluripotency markers (Supplementary Fig. 1). The ideal IM differentiation platform was first established by investigating three published protocols for directed differentiation of iPSCs into kidney progenitor cells^21–23^. To determine the efficiency of mesoderm induction, PCR and immunocytochemistry were used to assess expression of early mesoderm specific transcription factors *MIXL1* and *BRACHYURY*. Out of the three protocols tested, we found that 100 ng/ml of activin A with 3 μM of CHIR99021, a potent GSK-3b inhibitor and canonical WNT pathway agonist, rapidly and efficiently specified human iPSCs (line 87iCTR-n3) into mesoderm, which recapitulates Mae *et al*. (Fig. 1c)^21^. All three human iPSC lines showed a similar pattern, though the differentiation potential varied slightly between lines (Supplementary Fig. 2). The peak of *BRACHYURY* and *MIXL1* expression at day 2 followed by subsequent downregulation is consistent with the transient expression of these genes during gastrulation. In addition, BRACHYURY protein production showed a similar kinetic pattern during cell differentiation (Fig. 1d).

To optimize the efficiency of generating IM cells from mesoderm, we next examined the BMP signaling, which is known to regulate mesodermal cell type fate and further specification into IM^24, 25^. Importantly, BMP4 has been shown to regulate early ovarian follicle development and the creation of neonatal mouse uterine epithelium^26, 27^. While the Mae *et al*.^21^ protocol uses 100 ng/ml of BMP7 with 3 μM of CHIR99021, results here showed that exposure to 100 ng/ml of BMP4 with 3 μM of CHIR99021 provided a transient upregulation of *BRACHYURY* and *MIXL1* that is seen during gastrulation, which was not recapitulated with BMP7 treatment (Supplementary Fig. 3a). The combination of BMP4 and CHIR99021 also guided the optimal mesoderm differentiation into IM, which is shown by the expression levels of early IM markers *PAX2*, *OSR1* and *GATA3* (Fig. 1e). Once again, all three human iPSC lines showed a similar pattern of differentiation based on *PAX2* expression, with slightly varying levels of differentiation between lines (Supplementary Fig. 3b). Remarkably, BMP4-induced IM was more prone to differentiate towards FTE compared to BMP7, as suggested by the intermediate upregulation of markers primed for the female reproductive tract, namely *WT1* (Fig. 1e). In parallel, the induction of mesoderm and IM genes was complemented by the loss of pluripotency, as reflected by a reduction of expression of pluripotency genes *POU5F1, SOX2* and *TDGF1* (Fig. 1f). Notably, a minimal amount of endoderm and ectoderm was also derived, demonstrated by low expression of *SOX17* (endoderm) and *NCAM* (ectoderm) during differentiation (Supplementary Fig. 3c). Collectively, these data show that this differentiation protocol with BMP4 appears to be mostly specific for mesodermal linages and yielded the highest level of mesoderm and IM differentiation.

### Differentiation of Intermediate Mesoderm into Fallopian Tube Epithelia

**Figure 2:**
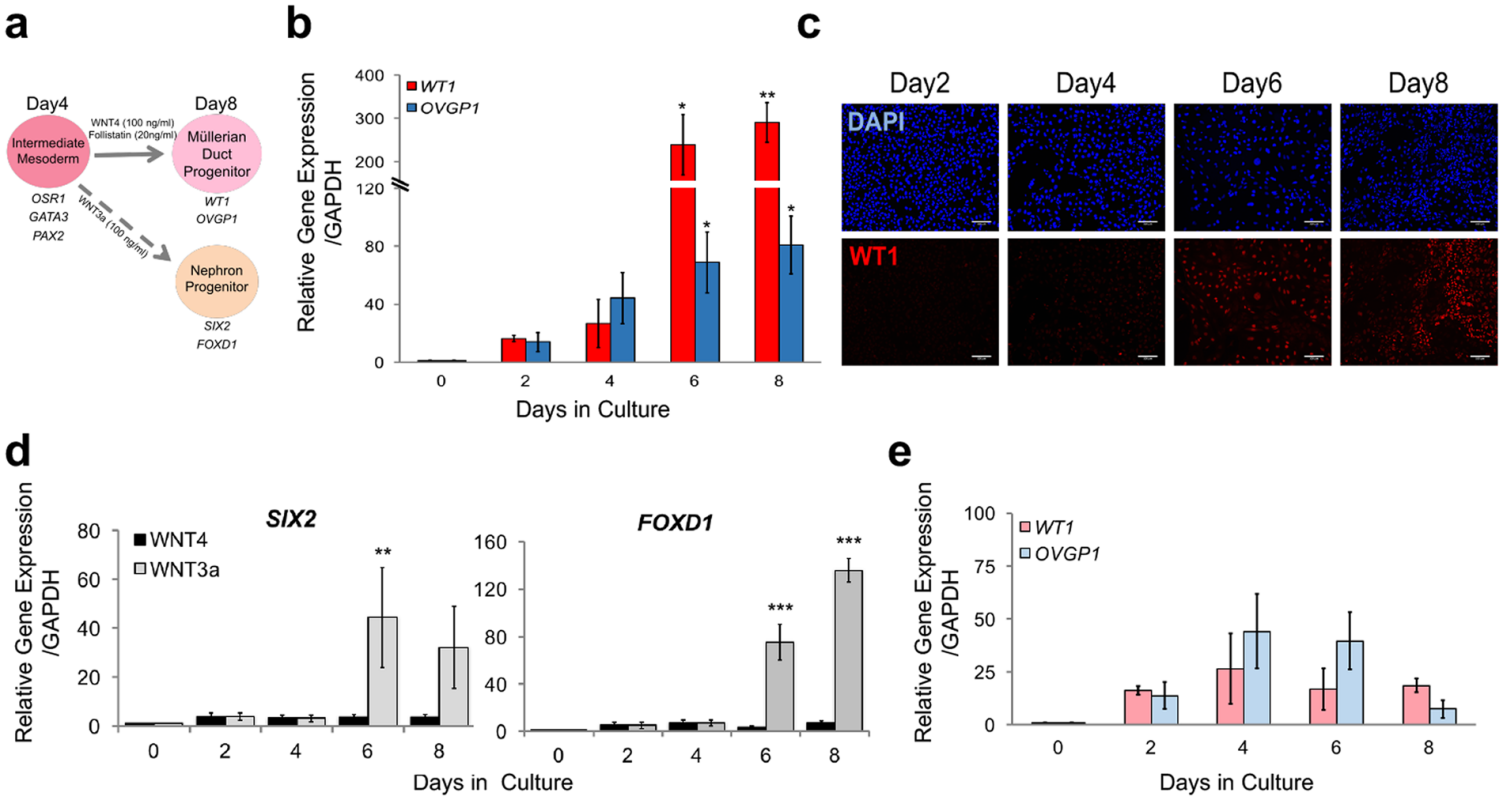
Differentiation of Intermediate Mesoderm into Fallopian Tube Epithelium Cells. (**a**) Timeline and factors involved in the differentiation of intermediate mesoderm into fallopian tube epithelium precursor cells. (**b**) qPCR quantification of gene expression kinetics for Müllerian duct markers, *WT1* and *OVGP1*, throughout Müllerian duct differentiation. (**c**) Immunocytochemistry demonstrating protein production and localization of WT1 during Müllerian duct differentiation. (**d**) mRNA fold change comparison of kidney markers *SIX2* and *FOXD1* in response to Müllerian (WNT4, black bars) vs nephric duct (WNT3a, gray bars) differentiation. (**e**) qPCR quantification of gene expression kinetics for Müllerian duct markers, *WT1* and *OVGP1*, throughout the nephric duct differentiation. Relative gene expression to iPSC stage (day 0) was calculated using the ΔΔCt method and normalized to endogenous *GAPDH* level for 87iCTR-n3 iPSC line. Error bars are SEM (n = 3 independent biological experiments). One way ANOVA with Holm-Sidak post-hoc test was used for this analysis, with significance at *p ≤ 0.05, **p ≤ 0.01, ***p ≤ 0.001.

The canonical WNT signaling pathway plays a crucial role in patterning the embryo during development and FTE formation. A subset of WNT gene family members including WNT3a and WNT4 are required for the initiation and elongation phases of Müllerian duct formation as well as for suppression of male differentiation pathways^28, 29^. To complete our FTE differentiation protocol, we defined the role of these WNT signaling in our *in vitro* model. Our result showed that cultures that were treated with 100 ng/ml of WNT4 on day 4 followed by 20 ng/ml of its downstream component Follistatin, which binds and bioneutralizes members of the TGF-beta superfamily, were more prone to differentiate into FTE (Fig. 2a). Müllerian duct differentiation was confirmed by both PCR and immunocytochemistry, which showed upregulated expression over time of the fallopian tube precursor markers *WT1* and *OVGP1* (Fig. 2b,c). Importantly, this protocol was specific to Müllerian duct differentiation, demonstrated by the lack of expression of the early kidney markers *SIX2*, *FOXD1*, and *PAX8* (Fig. 2d, Supplementary Fig. 4a, black bars and Supplementary Fig. 4b red channel), as well as lack of the intestinal and lung markers CDX2 and *NKX2-1*, respectively (Supplementary Fig. 5a,b). Interestingly, WNT4 along with prolonged CHIR99021 exposure led to the expression of early mesonephric duct markers *HOXB7* and *GATA3* (Supplementary Fig. 6a,b). However, compared to WNT4 alone (Fig. 2b), FTE markers *WT1* and *OVGP1* did not show increased expression in WNT4/CHIR99021 treatment (Supplementary Fig. 6c,d). Additionally, as with WNT4 alone (Fig. 2d), WNT4/CHIR99021 did not induce expression of kidney progenitor markers *SIX2* and *FOXD1* (Supplementary Fig. 6e,f). This suggests that continued CHIR99021 during WNT4 treatment might induce other WNT signaling factors that disrupt further Müllerian development.

Since Müllerian duct development occurs in parallel to the nephric duct, we also characterized the kinetics of kidney progenitor formation using our differentiation protocol. As in published protocols, treating cultures with WNT3A led to increased expression of the renal markers *SIX2*, *FOXD1* and *PAX8*, promoting the formation of the nephric duct and ureteric bud (Fig. 2d and Supplementary Fig. 4a, gray bars and 4c red channel)^21, 30^. In contrast, WNT3A treatment did not yield differentiated Müllerian duct, based on the nearly 10-fold decreased levels of *WT1* and *OVGP1* compared to WNT4 treatment in Fig. 2b (Fig. 2e). Collectively these results demonstrate that activation of WNT4 followed by Follistatin can selectively differentiate IM into FTE precursor cells.

### Generation of Human Fallopian Tube-like Organoid in 3D Culture

Remarkably, BMP4- and CHIR99021-treated cultures underwent morphogenesis that was similar to embryonic development. Between 3–5 days of treatment, flat cell sheets condensed into epithelial buds (Supplementary Fig. 7). Once harvested, these buds formed spheroids, which were placed into Matrigel beads along with pro-Müllerian growth factors. Since optimal IM formed at day 4, spheroids were collected on days 3 to 6. Day 4 was ideal for collection of spheroids, as cells collected on day 3 were immature and grew with unstable structure in Matrigel and spheroids collected after day 6 did not persist in culture (Supplementary Fig. 7). When these spheroids were grown in Matrigel that contained phenol red, they formed an organoid structure (Supplementary Fig. 8a). In contrast, when spheroids were grown in Matrigel not containing phenol red, they became branched and formed an unorganized matrix (Supplementary Fig. 8b). As phenol red is a weak estrogen mimic, these results indicate the significance of estrogen on FTE differentiation and maturation.

**Figure 3:**
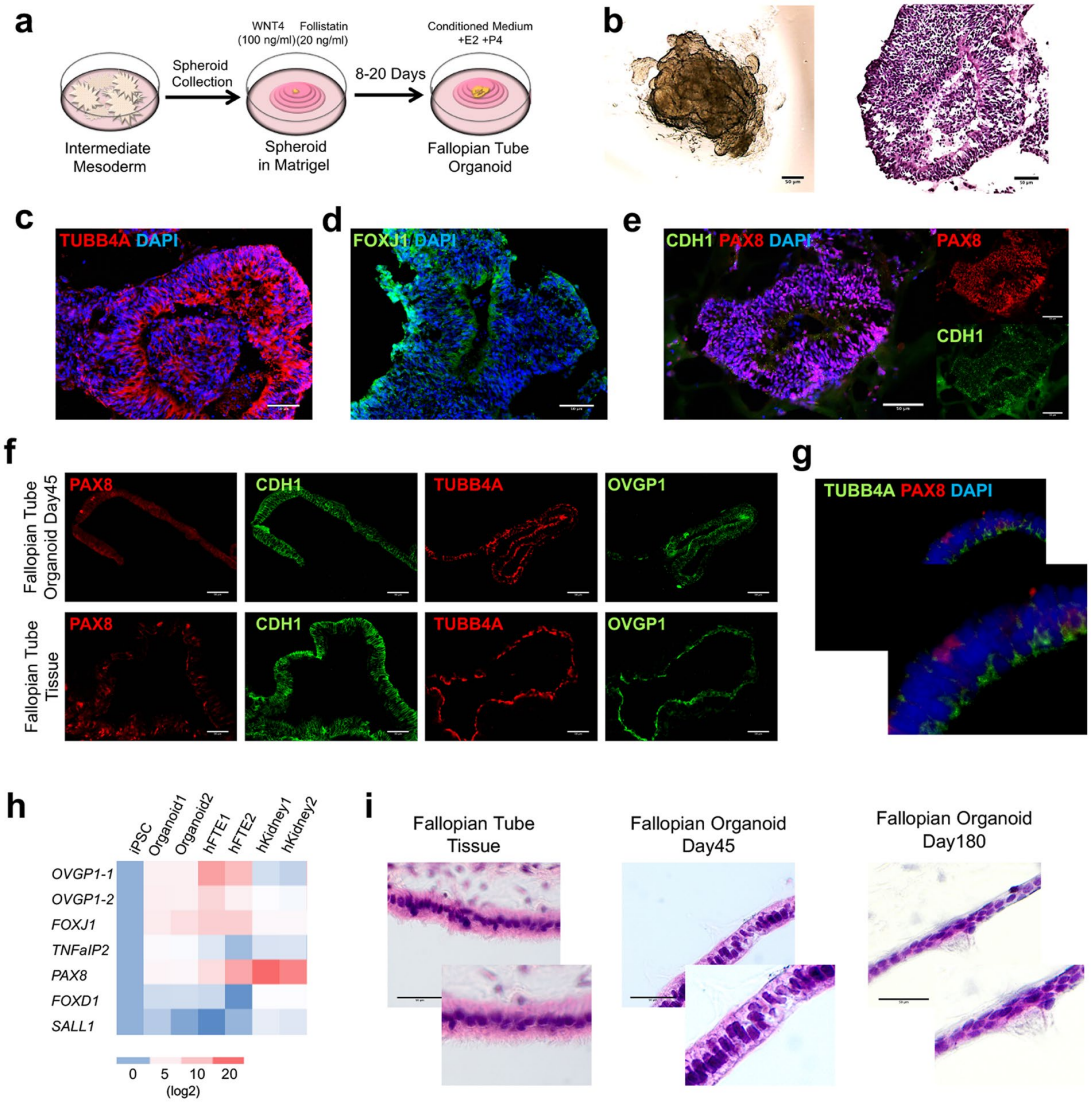
Development and Characterization of an iPSC-Derived Fallopian Tube Organoid. (**a**) Schematic of factors involved in the differentiation of fallopian tube organoids. (**b**) Bright field image and H&E staining of FTE organoid at day 14. (**c–e**) Immunocytochemistry for FTE markers TUBB4A, FOXJ1 and PAX8 and epithelial marker CDH1 (E-Cadherin) at organoid culture day 14. (**f**) Immunocytochemistry for FTE markers PAX8, TUBB4A, OVGP1 and epithelial marker CDH1 at FTE organoid culture day 45, along with human fallopian tube tissue. (**g**) Immunocytochemistry for FTE markers TUBB4A and PAX8 at FTE organoid culture day 45. (**h**) Gene expression of fallopian tube markers *OVGP1* (for 2 different primers*), FOXJ1, TNFaIP2* and *PAX8*, as well as kidney markers *SALL1* and *FOXD1* at organoids culture day 45, human fallopian tube and kidney. The color matrix of the heat map represents the log2(Ratio) of each individual gene relative to its expression at the iPSC stage. Relative gene expression to iPSC stage (day 0) was calculated using ΔΔCt method and normalized to endogenous *GAPDH* level for 87iCTR-n3 iPSC line (**i**) H&E staining of FTE organoid at culture day 45 and day 180, and human fallopian tube tissue.

While the estrogenic properties of phenol red initially improved organoid growth and organization, it was unable to sustain the organoids over longer periods. Steroid hormones, and estrogen in particular, are known to regulate development of the female reproductive tract, and estrogen has been shown to mediate cellular proliferation and differentiation during embryogenesis^31, 32^. Therefore, to increase the architectural complexity and gain the structure of the plicae, fallopian tube organoids were exposed to estrogen (E2) and progesterone (P4) (Fig. 3a). In addition, conditioned media from FTE cells freshly isolated from patient tissue was used to provide other unknown factors in the fallopian tube milieu that may be necessary for fallopian tube development.

This optimized protocol provided long-term organoids in matrigel that displayed luminal structures (Fig. 3a,b). Immunocytochemistry demonstrated that these fallopian tube structures contained ciliated (TUBB4A and FOXJ1) and secretory (PAX8) cellular components (Fig. 3c–e) and the lack of expression of kidney marker SIX2 in comparison to kidney progenitors (Supplementary Fig. 9a,b). However, these markers were found throughout the organoid, instead of in specific fallopian tube compartments as would be expected in mature FTE. In addition, there was no expression of the mature epithelial cell marker CDH1 (Fig. 3e).

### Progressive Maturation of Fallopian Tube Organoid in 3D Culture Over Time

To achieve functional maturation of FTE, the iPSC-derived FTE organoids were cultured in 3D Matrigel for an extended period with estrogen and progesterone supplemented media. Immunocytochemistry at day 30 showed that secretory cell marker PAX8 and ciliated cell marker TUBB4A, as well as the epithelial cell marker CDH1, exhibited similar expression patterns in FTE organoids model from two different iPSC lines (Supplementary Fig. 10), thereby demonstrating biological replicates between cell lines and technical replicates within the same cell line. Results at 45 days showed that the FTE ciliated marker, TUBB4A, and secretory cell markers, OVGP1 and PAX8 and the epithelial cell marker, CDH1, were now expressed in the expected sub-compartments seen in mature FTE, with TUBB4A being highly expressed along the lumen (Fig. 3f, Supplementary Fig. 11). The cellular phenotype and organization of the iPSC-derived FTE organoids were comparable to fresh human fallopian tube tissue (Fig. 3f). Importantly, mature organoids contained TUBB4A-positive and PAX8-negative ciliated cells as well as PAX8-positive secretory cells (Fig. 3g).

The differentiation of iPSC-derived organoids into fallopian tube mature cells over time was further demonstrated using heat map analysis, which showed increased expression of the ciliated cell marker *FOXJ1* and secretory cell markers OVGP1and PAX8, which were similar to human fallopian tube tissue (Fig. 3h). Critically, iPSC-derived FTE organoids not only expressed both ciliated and secretory cell markers but also formed visible cilia, further demonstrating that this novel model closely mimics the proper physiology and anatomy of the human FTE (Fig. 3i).

## Discussion

Human iPSCs are derived using technologies to reprogram adult cells back to a pluripotent state with the subsequent potential to differentiate into any cell type in the human body. Reprogramming to human iPSCs possesses the essential advantages of eliminating the requirement for embryonic material while allowing for the generation of pluripotent cells with known genetic factors in a patient-specific manner. Moreover, combined with 3D cell culture techniques, 3D iPSC-derived organoid models can be developed to reconstitute features of organs by mimicking more closely the complex cellular heterogeneity and cell-cell/cell-matrix interactions, thereby overcoming many of the limitations of traditional monolayer cell culture systems.

Intermediate mesoderm generates several components of the urogenital system, including reproductive tracts and also the kidneys, the gonads, and their respective duct systems. Priming the IM into only the desired cell fate has been a challenge for the iPSC-derived organoid model. To our knowledge, all established iPSC-derived IM platforms have been developed to model the kidney formation using the combination of WNT, Nodal/Activin, and BMP signaling^21–23^. In this study, different WNT signaling was modulated to successfully develop a novel differentiation protocol with key components to drive Müllerian duct and the female reproductive tract development rather than kidney. The most efficient differentiation platform to mimic embryonic fallopian tube development used CHIR99021 and activin A, along with BMP4 that was discovered to be critical for conversion of IM into female reproductive tract or Müllerian duct. To our knowledge, this study presents the first iPSC-derived FTE *in vitro* model. We have demonstrated direct differentiation of human iPSCs into fallopian tube-like precursor cells and, moreover, established iPSC-derived human fallopian tube organoids that recapitulate the complex 3D architecture of FTE tissue.

During embryogenesis, WNT signaling pathways play a prominent role in regulating cell fate specification and in determining cell polarity and migration. WNT signaling is often correlated with cell proliferation and tissue repair after acute injury. However, recent human FTE organoid models with bi-potent FTE stem cells indicated the function of WNT3A in fallopian epithelial renewal and the regulation of stemness, suggesting the distinct role of WNT pathways in embryonic development and adult stem cell function^33^. The current study found that exposure to WNT4, instead of WNT3A, and activation of the downstream effector Follistatin were vital to specify IM differentiation into Müllerian duct and an FTE cell fate *in vitro*. Interestingly, we also showed that subsequent activation of WNT3A rather than WNT4 promoted nephric duct formation, which reflects the different molecular ability of WNTs to regulate specific cell fates.

Many fundamental cellular processes are differentially regulated between 2D and 3D cultures^8, 34, 35^. Exposure of differentiated iPSCs to a 3D growth platform enabled them to self-organize into luminal structures that model fallopian tube structure with secretory and ciliated cell components. Critically, the exposure to primary fallopian tube epithelial conditioned media is required for maintenance of 3D FTE luminal structure and architecture, indicating that there are unknown factors responsible for the organization of cellular structure of the fallopian tube.

In this study, we generated the basis of a platform for directed differentiation of iPSCs into fallopian tube epithelial cells, however, there is room for further improvement with regard to long-term culturing and functional maturity. Growth and successful cultivation may depend on different cell-matrix interactions and other growth factors that need to be further studied.

iPSC-based *in vitro* models of cancer can help to understand pathological processes at the molecular and cellular level. In addition, they potentially provide a critical platform to study drug resistance and to develop novel therapies. Importantly for ovarian cancer, iPSC-derived fallopian tube organoids can provide a faithful human cellular model to investigate the fallopian tube origin of serous carcinogenesis in ovarian cancer and to explore early cancer pathogenesis and progression. This platform can also be used study germline mutations that affect ovarian cancer development and to identify the critical steps and the sequence of genetic alterations involved in high grade serous carcinogenesis. In summary, iPSC-derived fallopian tube epithelium provides a powerful *in vitro* model of ovarian cancer that can recapitulate early *de novo* genomic alterations, faithfully model disease progression and ultimately uncover novel treatments.

## Methods

### Ethics Statement

All human samples were obtained using the approved IRB in accordance with relevant guidelines and regulations under IRB PRO00000901 (The Women’s Cancer Program Tissue Bank) protocol number, and informed consent was acquired from all human subjects and/or their legal guardians. Human B-cells, mammary epithelial cells, and fibroblast cells were obtained from the Institute for Medical Research. The Cell Repository maintains the informed consent and privacy of the donor. All the cell lines and protocols in the present study were used in accordance with the guidelines approved by the stem cell research oversight committee (SCRO) and institutional review board (IRB) under the auspice IRB-SCRO Protocols Pro00032834 (iPSC Core Repository and Stem Cell Program) and Pro00021505 (Svendsen Stem Cell Program).

### Data Availability

All data generated or analyzed during this study are included in this manuscript (and its Supplementary Information files).

### iPSC Culture

Human B-cells, mammary epithelial cells, and fibroblasts were used to derive iPSCs, which were cultured in mTeSR®1 medium (STEMCELL) on growth factor-reduced Matrigel™ Matrix (BD Biosciences)-coated plates at 37 °C in a 5% CO_2_ incubator. Briefly, 70–90% confluent human iPSC colonies were dissected into small squares using the EZ Passage tool (Invitrogen). For weekly passaging, colonies were lifted carefully with a cell scraper, removed using a 5 ml glass pipette, and replated at a 1:6 ratio. All the cell lines were tested for mycoplasma contamination monthly.

### Directed Differentiation of iPSCs in Chemically Defined Conditions

Human iPSCs were split onto Matrigel-coated plates and cultured in mTeSR®1 medium until 80% confluent. Three intermediate mesoderm protocols were compared, derived from Mae *et al*.^21^, Xia *et al*.^3^, and Takasato *et al*.^22^. The final protocol that achieved the best efficiency and results for creating fallopian tube epithelium was a modified Mae *et al*.^21^ protocol, as defined below^21^.

### Day 0–2

Cells were exposed to 100 ng/ml human recombinant activin A (Stemgent) and 3 μM CHIR99021 (Cayman Chemicals) to differentiate towards mesoderm, and cultured in DMEM/F12 (Gibco) +Glutamax (Invitrogen) supplemented with 500 U/ml penicillin streptomycin (Gibco) and 2% fetal bovine serum (FBS) with addition of 10 μM ROCK inhibitor Y-27632 (Stemgent).

### Day 2–4

To differentiate cells towards IM, media was changed to DMEM/F12 (Gibco) +Glutamax (Invitrogen) supplemented with 0.1 mM non-essential amino acids (Invitrogen), 500 U/ml penicillin/streptomycin (Gibco), 0.55 mM 2-mercaptoethanol, 10% Knockout Serum Replacement (Invitrogen), 100 ng/ml BMP4 (R&D Systems), 3 μM CHIR99021 (Cayman Chemicals), and 10 μM ROCK inhibitor Y-27632 (Stemgent).

### Day 4–6

Spheroids were collected from wells and re-plated per methods described below. To differentiate the spheroid cultures towards Müllerian epithelium, media was changed to Fallopian tube media (FTM) containing DMEM/F12 (Gibco) +500 U/ml penicillin/streptomycin (Gibco), and 2% reconstituted Ultroser G (15950-017, Pall) and 10 μM ROCK inhibitor Y-27632 (Stemgent). For FTE differentiation, 100 ng/ml human recombinant WNT4 (R&D Systems) with or without 3 μM CHIR99021(Cayman Chemicals), 100 ng/ml human recombinant WNT3A (R&D Systems) with or without 3 μM CHIR99021 (Cayman Chemicals) were added.

### Day 6–8

FTM was changed and 20 ng/ml human recombinant Follistatin (Peprotech), 1 ng/ml estrogen and 33 ng/ml progesterone were added.

### Growing FTE Organoids from Spheroids in Matrigel

Spheroids were collected on day 4 from every well under a stereomicroscope using a 200 μl barrier pipette tip and pooled into a 1.5 ml microcentrifuge tube. Spheroids were then mixed with 50 μl Matrigel (BD Biosciences) containing estrogen (1 ng/ml) and progesterone (33 ng/ml), and slowly pipetted into the middle of one well of a 24-well Nunclon Delta surface dish using previously described method by McKracken *et al*.^36^. The 3D droplet was allowed to solidify for 10–15 minutes in a tissue incubator, and Matrigel beads were then bathed in FTM supplemented with the same concentration of growth factors. Media was replaced every 3–4 days as necessary, and cells were replated every two weeks. All the cultures were tested for mycoplasma contamination monthly.

### RNA isolation and real-time PCR analysis

Total cellular RNA was isolated using Qiagen RNeasy Mini kit following manufacturer recommendations (Qiagen). RNeasy-treated total RNA (1 μg) was used for cDNA synthesis using the Quantitect Reverse Transcription Kit for cDNA synthesis for PCR (Qiagen). Real-time PCR was performed using the SYBR Green Supermix (BioRad). The levels of expression of respective genes were normalized to corresponding *GAPDH* values and shown as fold change relative to the value of the control sample. All sample analyses were carried out in triplicate. List of primers used for real-time PCR experiments are listed in S2 Table. At least one set of replicate was performed blindly.

### Immunocytochemistry for Monolayer Culture

Monolayer cultures were grown on poly-l-lysine and ornithine coated glass coverslips. Cells were washed once with phosphate-buffered saline (PBS) and fixed with 4% paraformaldehyde (PFA) in 1X PBS for 20 minutes and permeabilized in PBS containing 0.5% Triton X-100 (PBS-T) (Sigma) for 5 minutes at room temperature. Cells were then blocked with 10% FBS in PBS for 1 hour at room temperature, followed by a 2-hour incubation with primary antibodies (see section below). The cultures were washed with PBS-T three times for 15 minutes each at room temperature and incubated with species-specific AF488 or AF594-conjugated secondary antibodies followed by nuclei counterstain with DAPI. Following three washes in PBS-T, the coverslips were mounted onto glass slides and imaged using Nikon/Leica microscopes. Each selected image is representative of a minimum of three independent experiments with at least two technical duplicates.

### Immunocytochemistry for Organoid and Fallopian Tube Tissue

Organoids and fallopian tube tissues were fixed with 4% PFA in 1X PBS for 20 minutes, followed by three PBS washes. The fixed organoids and fallopian tube tissues were then sunk in 30% sucrose at 4 °C overnight and then embedded in OCT (Tissue-Tek). Frozen sections were collected at 12 μm using a cryostat onto glass slides and stored at −80 °C. Each section was rehydrated with 1X PBS for 5 min and blocked in a solution of 10% FBS in PBS + 0.05% Triton X-100 (PBS-T) for 1 hour at room temperature, followed by 2 hours incubation at room temperature in primary antibodies (see section below) in blocking solution. The slides were washed with PBS-T three times for 15 minutes each at room temperature and incubated with species-specific AF488 or AF594-conjugated secondary antibodies followed by DAPI counterstain. Following three washes in PBS-T, the tissue was covered with a glass slide and imaged using Nikon/Leica microscopes. Each image selected for figures of this manuscript is representative of a minimum of three independent experiments with at least two technical duplicates.

### Antibodies

The following primary antibodies were used for fluorescence microscopy experiments at 1:200 dilution and secondary antibodies at 1:400 dilution: WT1 (Abcam, ab89901), PAX8 (Proteintech, 21384-1-AP), TUBB4A (Abcam, ab1315), BRACHYURY (Abcam, ab20680), POU5F1 (Stemgent, 09-0023), Nanog (Stemgent, 09-0020), SOX2 (Stemgent, 09-0024), TRA-1-60 (Stemgent, 09-0010), TRA-1-81 (Stemgent, 09-0011), SSEA4 (Stemgent, 09-0006), CDX2 (Biocare Medical, CM226A), SIX2 (Proteintech, 11562-1-AP), FOXJ1 (Abcam, ab40869), CDH1 (R&D System, AF648), OVGP1 (SIGMA, HPA062205) and DAPI (Molecular Probes, D3571).

### Fallopian Tube Tissue Collection

Fallopian tubes epithelial cells were collected from patients undergoing surgery for benign gynecological indications, such as adnexal mass, fibroids, or other benign conditions not affecting the fallopian tubes. Tissues were inspected and confirmed by pathology to be healthy and not associated with gynecologic malignancy. Upon surgical excision, fallopian tubes were collected in warmed sterile FTM as defined above. Fallopian tubes were rinsed 2 times for 10 mins with Red Blood Cell lysis buffer (0.144 M NH_4_Cl and 0.014 M NH_4_HCO_3_ in 10:1 ratio).

### Statistical Analysis

Statistical analyses were performed by using Prism software (GraphPad Software, La Jolla, California). All quantitative data were expressed as mean values ± Standard Error of the Mean (SEM) and analyzed by analysis of variance (ANOVA) followed by a Holm-Sidak analysis of mean differences in three biological replicates. Differences were considered significant at *p ≤ 0.05, **p ≤ 0.01, and ***p ≤ 0.001. Data sets were summarized with descriptive statistics including F value and degree of freedom in S3 and S4 Tables^31–36^.

## Electronic supplementary material

Supplementary Figures

Supplementary Table3

Supplementary Table4
