# Supplementary material for: Directed Differentiation of Human Induced Pluripotent Stem Cells into Fallopian Tube Epithelium
Source: Sci Rep. 2017 Sep 6;7:10741. doi: 10.1038/s41598-017-05519-2 (PMC5587694; doi:10.1038/s41598-017-05519-2)
Supplement: Supplementary file 1 — Supplementary Figures [file 41598_2017_5519_MOESM1_ESM.pdf]

Directed Differentiation of Human Induced Pluripotent Stem Cells into Fallopian Tube Epithelium

Nur Yucer[1,2], Marie Holzapfel[2], Tilley Jenkins Vogel[2], Lindsay Lenaeus[1], Loren Ornelas[1], Anna Laury[3], Dhruv Sareen[1], Robert Barrett[1], Beth Y. Karlan[2,4,*] and Clive N. Svendsen[1,4,*]

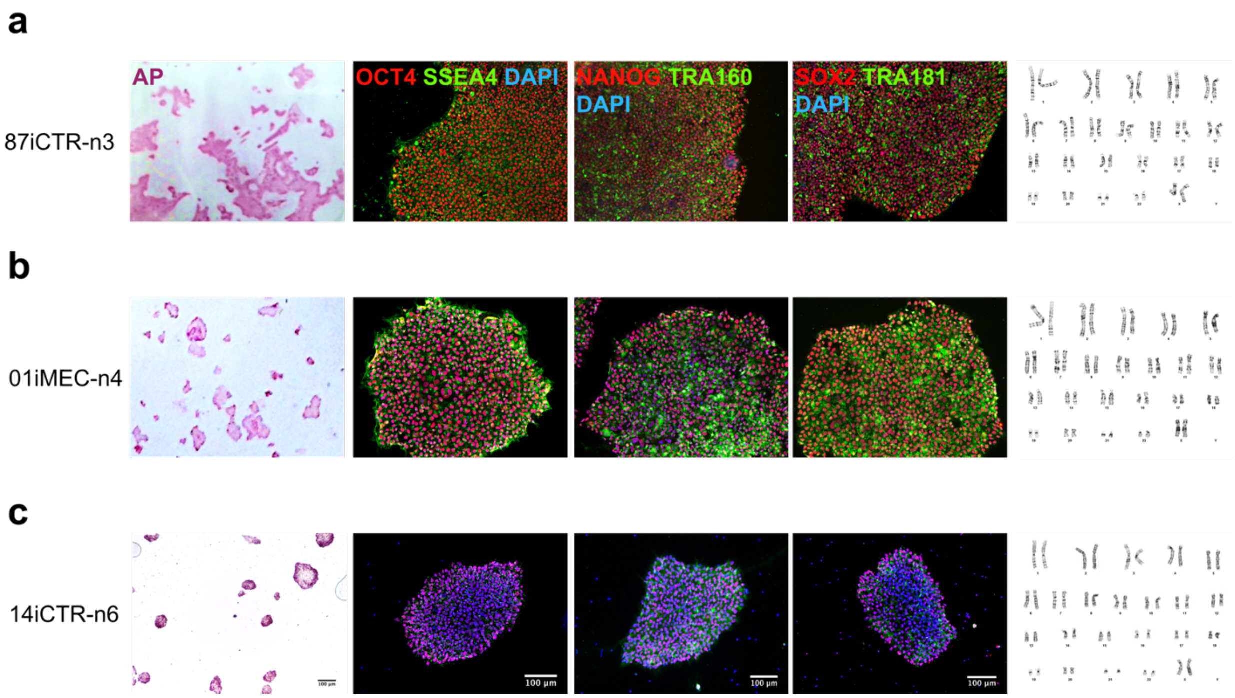

Supplementary Figure 1: Characterization of iPSC lines. All characterized iPSC lines (87iCTR-n3, 01iMEC-n4 and 14iCTR-n6) express pluripotent stem cell markers a) Alkaline Phosphatase (AP) and b) for OCT4, NANOG, SOX2, TRA160, TRA181, SSEA4 based on immunocytochemistry, as well as c) exhibit a normal karyotype based on G-band analysis.

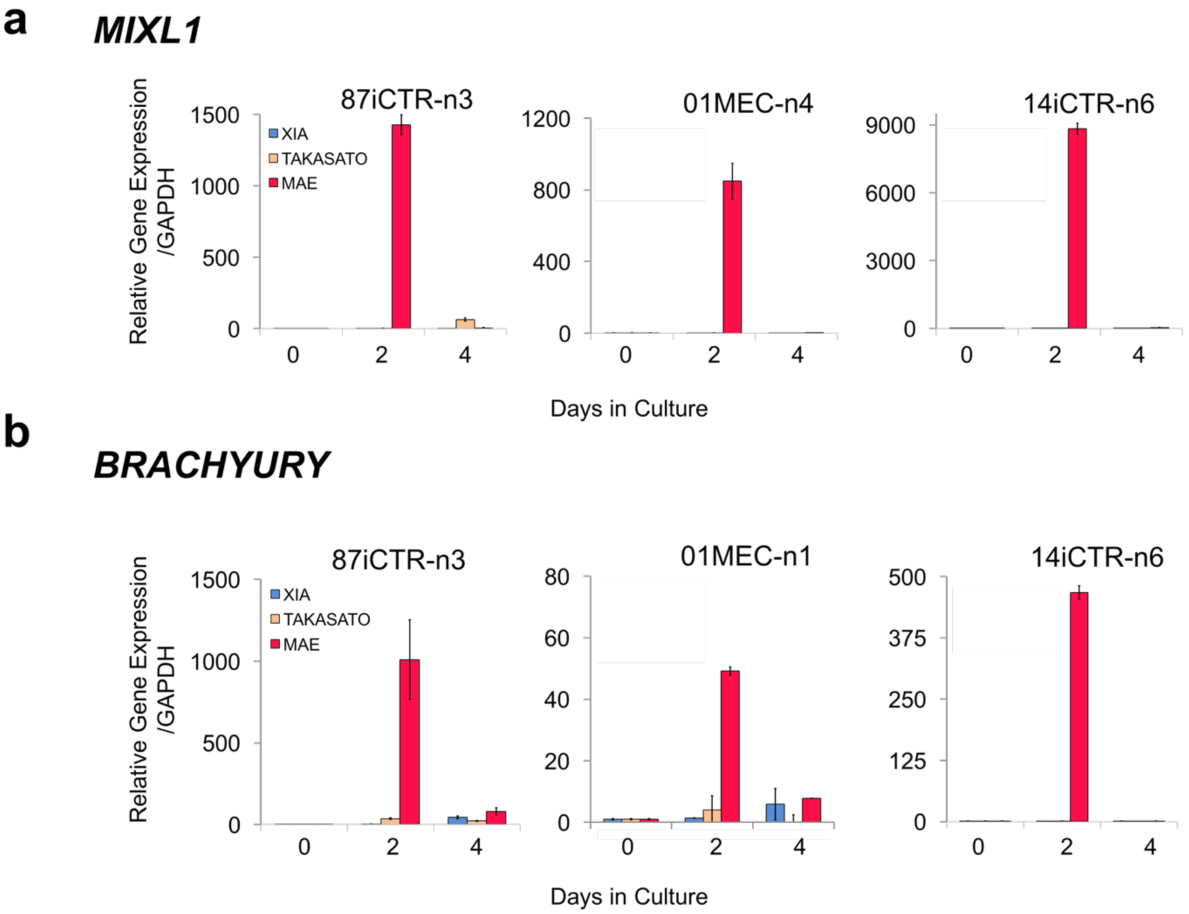

Supplementary Figure 2: IM differentiation efficiency for established protocols. Expression kinetics of mesoderm markers a) *MIXL1*- and b) *BRACHYURY* during the 6-day differentiation course of iPSC lines 87iCTR-n3, 01iMEC-n4 and 14iCTR-n6 using protocols from Xia et. al. (2013), Mae et. al. (2013), Takasato et. al. (2014)[21-23].

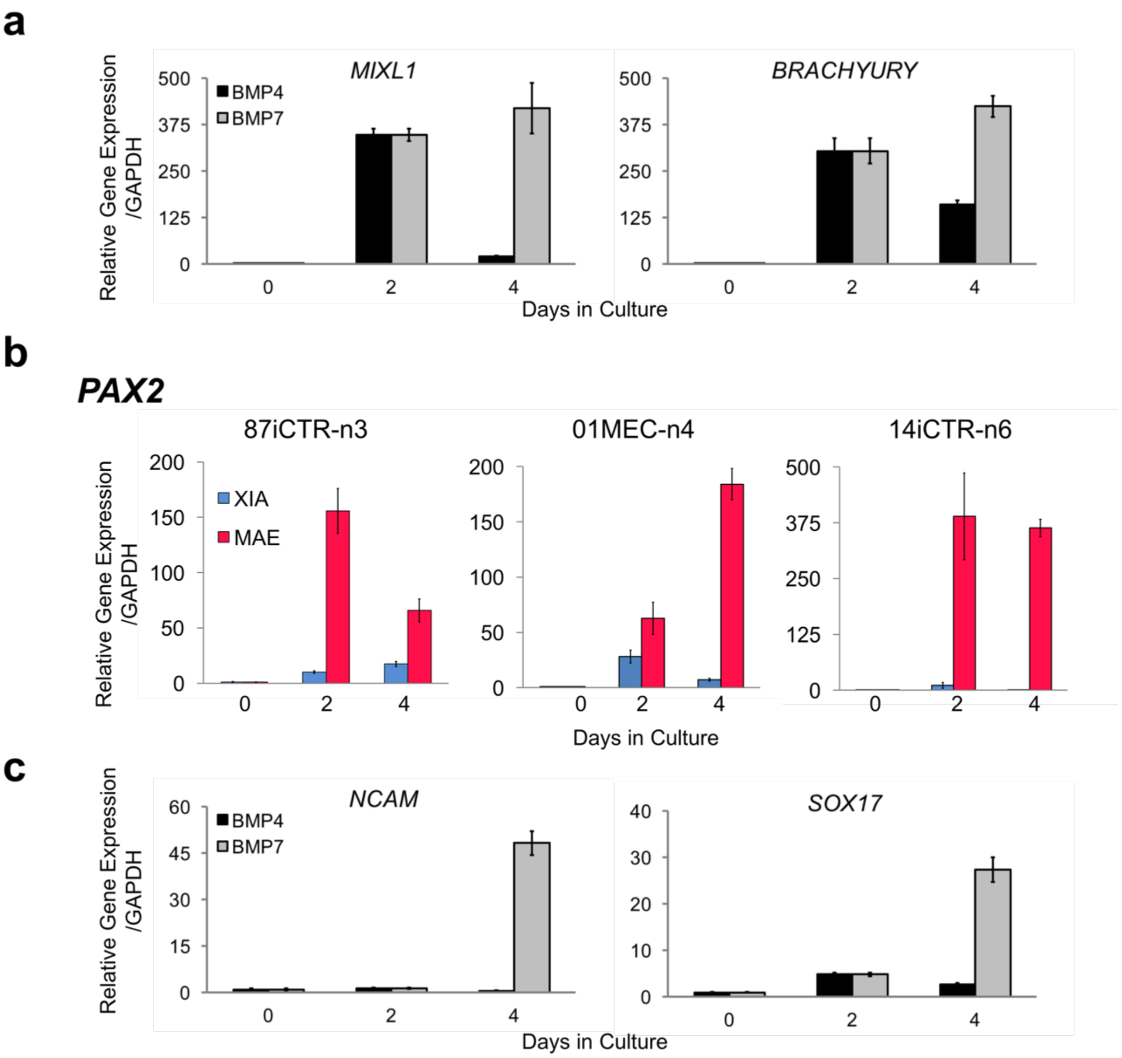

Supplementary Figure 3: Substitution to Mae et al. protocol with BMP4 from BMP7.

a) Expression kinetics of mesoderm markers *MIXL1*- and *BRACHYURY* during differentiation with BMP4 vs BMP7 treatment for 87iCTR-n3 iPSC line. b) Expression of intermediate mesoderm marker, *PAX2,* during the 6-day differentiation course of iPSC lines 87iCTR-n3, 01iMEC-n4 and 14iCTR-n6 using protocols from Xia et. al. (2013) and Mae et. al. (2013)[21-23]. c) Expression kinetics of ectoderm marker NCAM and endoderm marker SOX17 during differentiation with BMP4 vs BMP7 treatment for 87iCTR-n3 iPSC line.

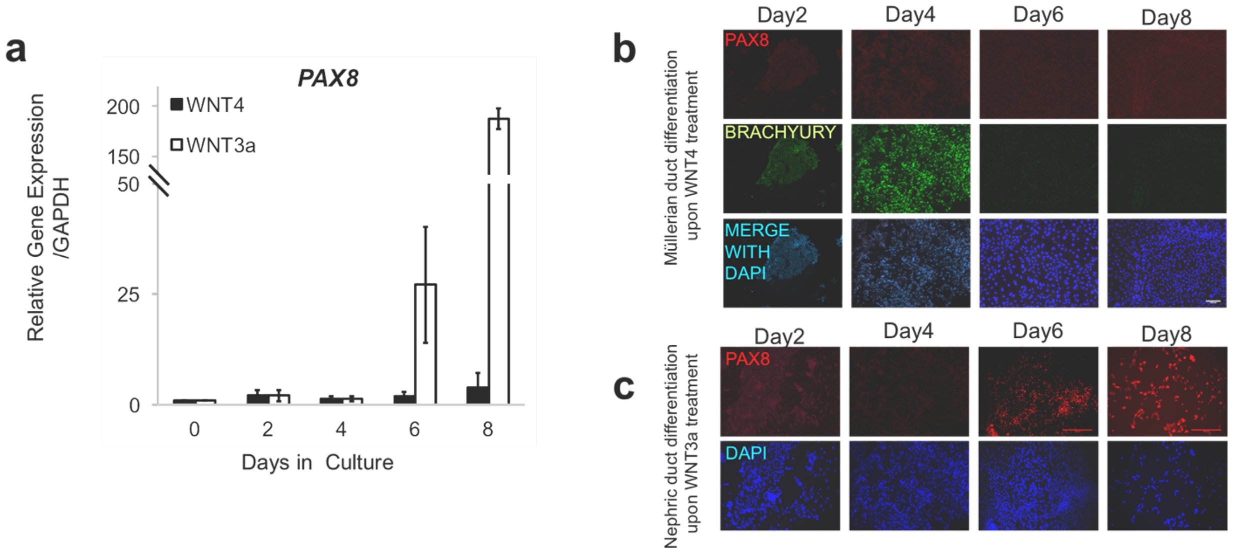

Supplementary Fig 4. PAX8 expression pattern during the course of differentiation: a) qPCR quantification of gene expression kinetics for PAX8 in response to Müllerian (WNT4, black bars) vs nephric duct (WNT3a, gray bars) differentiation. b) Immunocytochemistry demonstrating PAX8 expression during Müllerian duct differentiation. c) Immunocytochemistry demonstrating protein production and localization of PAX8 during nephric duct differentiation. d) Immunocytochemistry for PAX8 at FTE organoid culture day, day 30 and human fallopian tube tissue. e) Gene expression of PAX8 at organoids culture day 45, human fallopian tube and kidney. The color matrix of the heat map represents the log2(Ratio) of PAX8 to its expression at the iPSC stage. Relative gene expression to iPSC stage (day 0) was calculated using ΔΔCt method and normalized to endogenous GAPDH level for 87iCTR-n3 iPSC line.

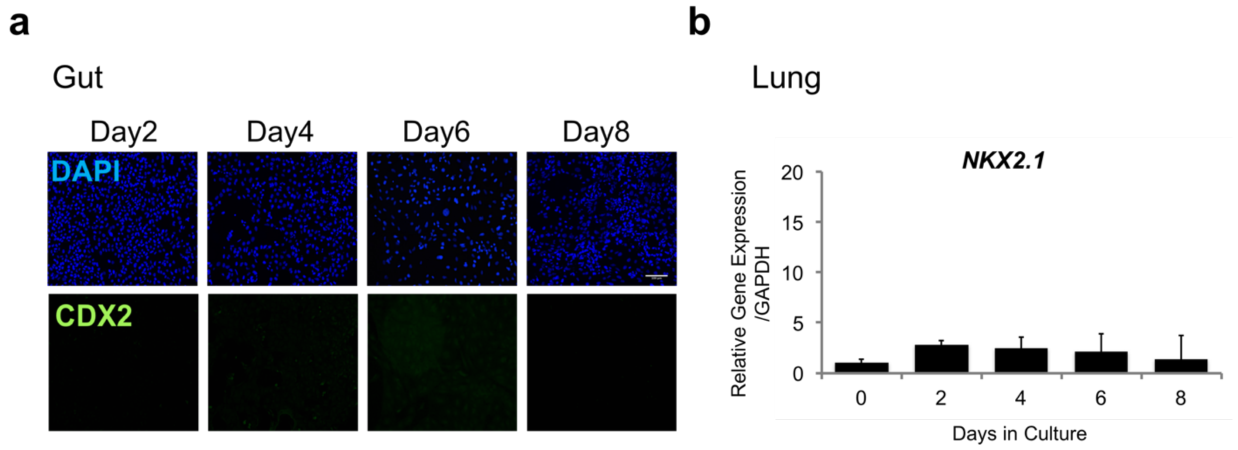

Supplementary Figure 5: Immunocytochemistry analysis throughout the Müllerian duct differentiation for production of a) gut marker CDX2 and b) lung marker *NKX2.1*.

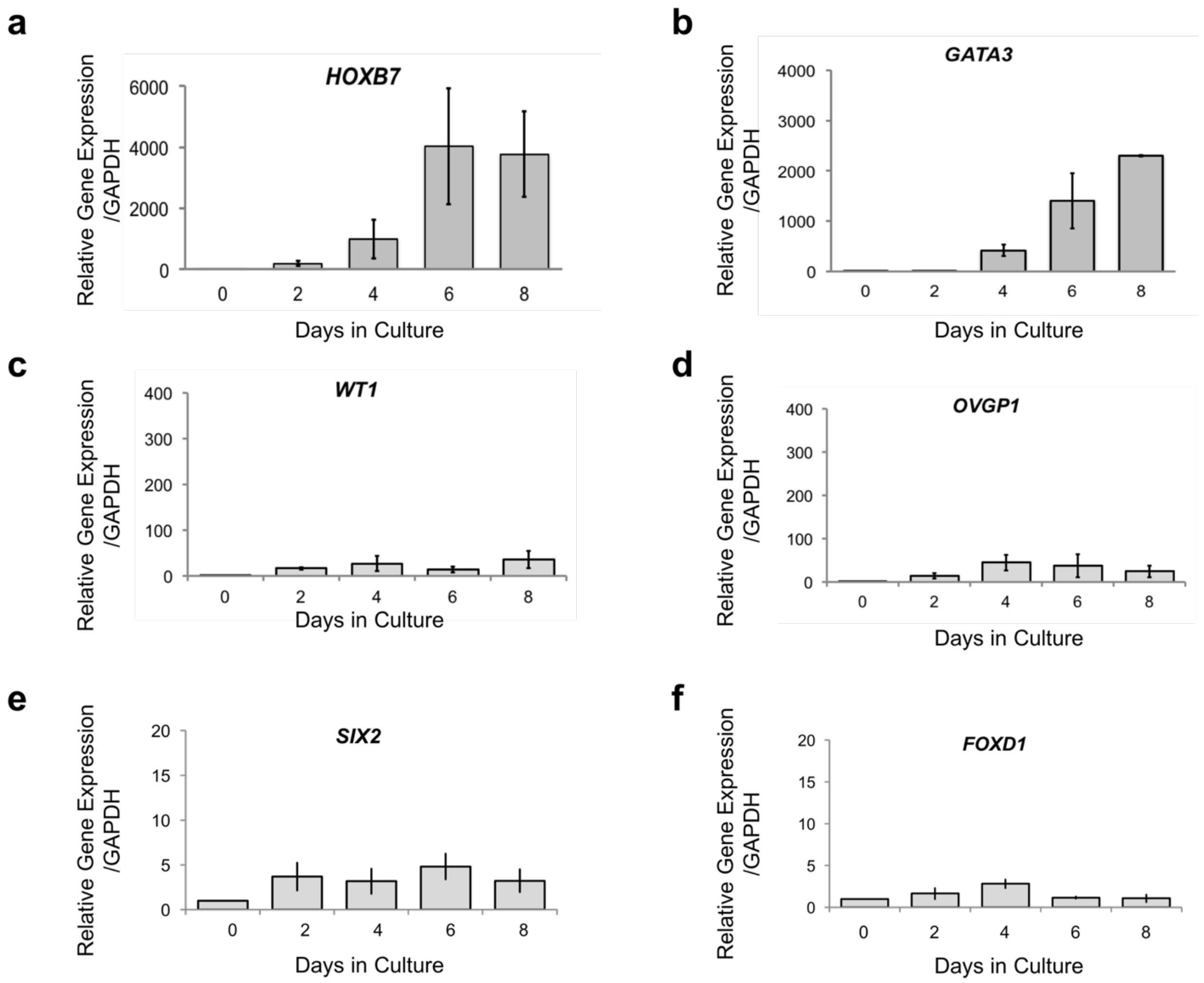

Supplementary Figure 6: Expression kinetics of a,b) early mesonephric duct markers *HOXB7* and *GATA3,* c,d) Müllerian duct markers *WT1* and *OVGP1* and e,f) kidney markers *SIX2* and *FOXD1* with WNT4/CHIR99021 treatment of the 87iCTR-n3 iPSC line.

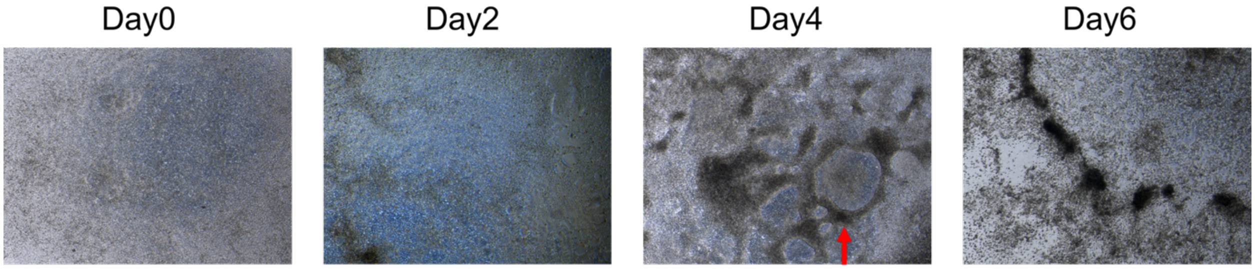

Supplementary Figure 7: Bright field image of Müllerian duct differentiation in monolayer culture for 6 days. Red arrow indicates the epithelial bud formation at day4.

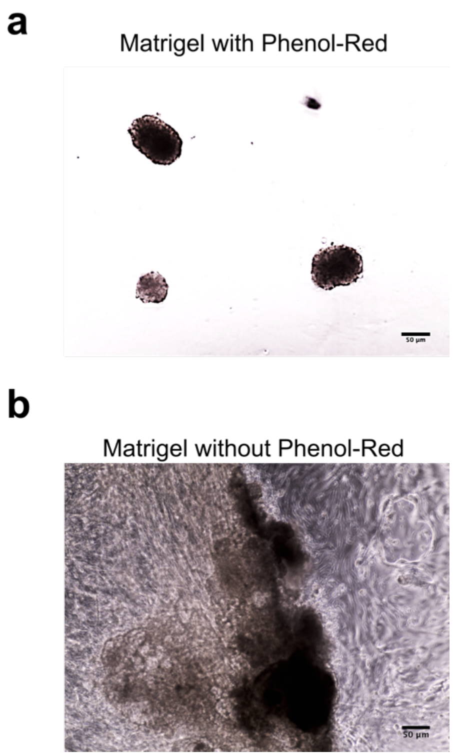

Supplementary Figure 8: Bright field image of 3D structure of FTE organoid at day 14 in a) Matrigel with Phenol Red vs b) Matrigel without Phenol Red.

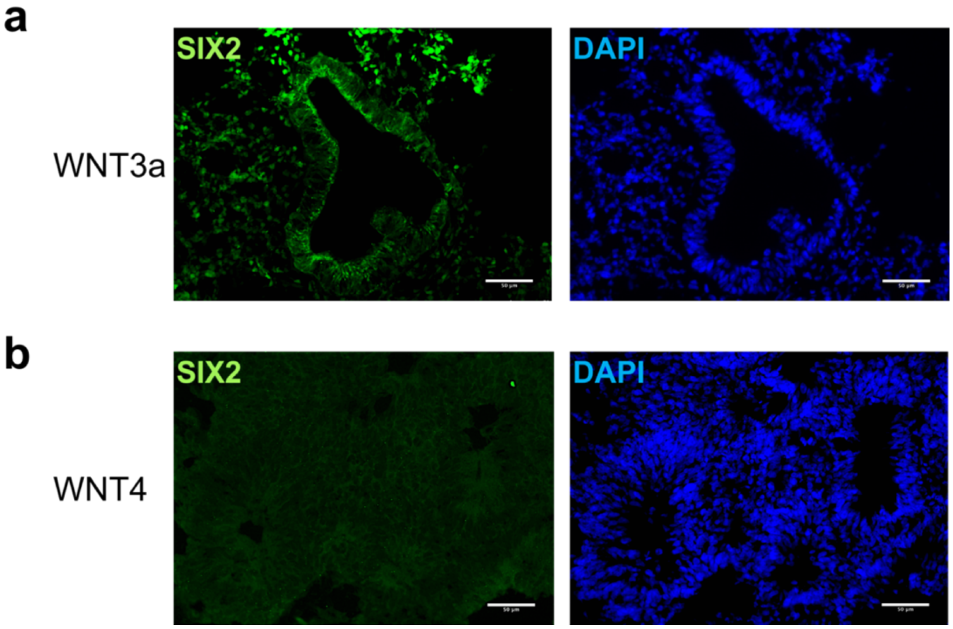

Supplementary Figure 9: Immunocytochemistry analysis of day 21 organoid for kidney marker SIX2 with a) WNT3A treatment as a positive control for kidney differentiation compared with b) WNT4 treatment for Müllerian duct differentiation.

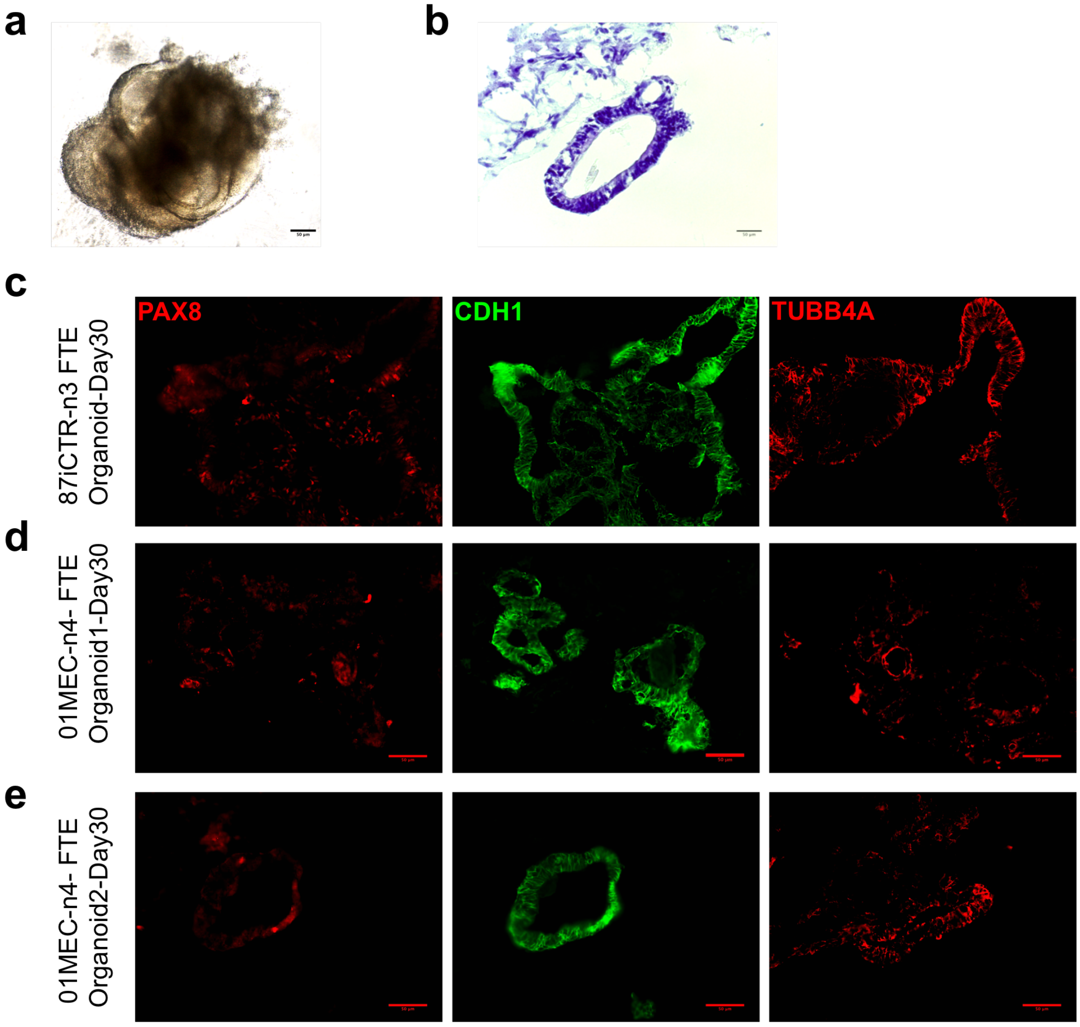

Supplementary Figure 10: a) Bright field image of 3D structure of FTE organoid from 01iMEC-n4 iPSC lines at day 30 in. b) H&E staining of FTE organoid from 01iMEC-n4 iPSC lines at day 30. (c-e) Immunocytochemistry for FTE markers TUBB4A and PAX8, and epithelial marker CDH1 (E-Cadherin) at organoid culture day 30 , 87CTR-n3 iPSC, 01iMEC-n4 iPSC biological replicate1 and 01iMEC-n4 iPSC biological replicate2 respectively.

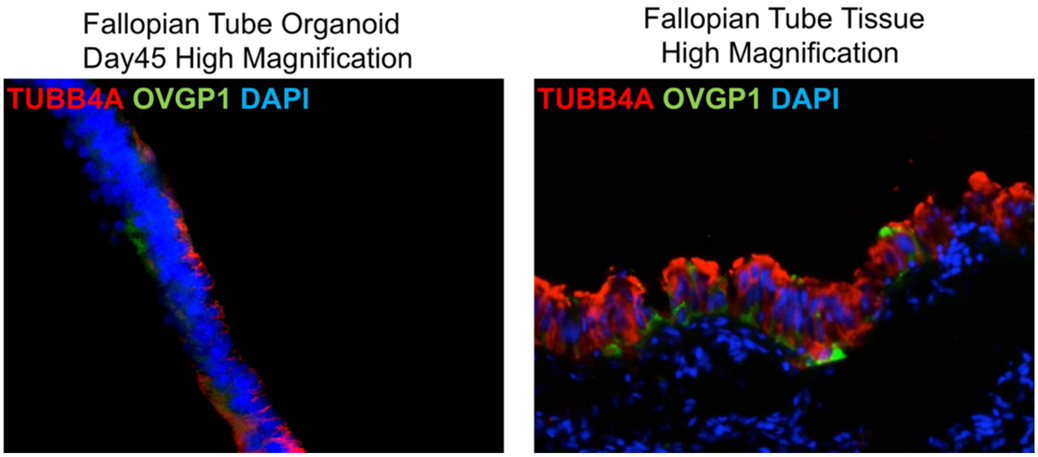

Supplementary Figure 11. Development and Characterization of an iPSC-Derived Fallopian Tube Organoid. Immunocytochemistry for FTE markers TUBB4A and OVGP1 at FTE organoid culture day 45, along with human fallopian tube tissue in high magnification.

Cell ID	Parent Tissue	Biopsy	Gender	Karyotype
87iCTR-n3	EBV B-Cell	Blood	Female	Normal
01iMEC-n4	Mammary Epithelial Cells	Mammary Epithelial	Female	Normal
14iCTR-n6	Fibroblast	Skin	Female	Normal

Supplementary Table 1. Cell background information. Cell line description, parent tissue, biopsy, gender, and karyotype.

Primer	Forward	Reverse
BRACHYURY	GCTGTGACAGGTACCCAACC	CATGCAGGTGAGTTGTCAGAA
FOXD1	GACTCTGCACCAAGGGACTG	CAATTGGAAATCCTAGCAGTAAAGT
FOXJ1	GGGGTGGGAGCAACTTCT	CCTCCTCCGAATAAGTATGTGGT
GAPDH	GTGGACCTGACCTGCCGTCT	GGAGGAGTGGGTGTCGCTGT
GATA3	CTCATTAAGCCCAAGCGAAG	GTCTGACAGTTCGCACAGGA
HOXB7	CCGAGAGTAACTTCCGGATCTA	CGTCAGGTAGCGATTGTAGTGA
MIXL1	GGTACCCCGACATCCACTT	GCCTGTTCTGGAACCATACCT
NCAM	GATTCCTCCTCCACCCTCAC	CAATATTCTGCCTGGCCTGG
NKX2.1	TCATTTGTTGGCGACTGG	TGCTTTGGACTCATCGACAT
OSR1	GGACCTCTGCGGAACAAG	TGCAGGGAAGGGTGGATA
OVGP1	AAGCTGTTGCTGTGGGTTG	TGTGCCCAGTTGGTGAAAT
OVGP1-2	AATTCTCTACCCAGAGTTCAACAAA	CCGATGGACAGTAGTGTTTTCA
PAX2	GAAGTGCCCCCTTGTGTG	TCGTTGTAGGCCGTGTACTG
POU5F1	ACCCACACTGCAGCAGATCA	CCACACTCGGACCACATCC
SALL1	ATTGCAGCCTAGCCAAAAAG	ACCAGCTGAGCAGAAAGGTC
SIX2	CAGGTCAGCAACTGGTTCAA	AGCTGCCTAACACCGACTTG
SOX17	ACGCCGAGTTGAGCAAGA	TCTGCCTCCTCCACGAAG
SOX2	GGGGGAATGGACCTTGTATAG	GCAAAGCTCCTACCGTACCA
TDGF1	AGGGAACAATGACAGAGTGTGA	CCCGAGATGGACGAGCAAAT
TNFaIP2	GAGCCACGGCTTTGACAC	GTGCGTGAACCTCTTGAACA
WT1	GAATGCATGACCTGGAATCA	TCTGCCCTTCTGTCCATTTC

Supplementary Table 2: Primers List

Supplementary Table 3 and 4: Statistical Analysis

Provided as Excel File
